# Comparison of seasonal variation in the fasting respiratory quotient of young Japanese, Polish and Thai women in relation to seasonal change in their percent body fat

**DOI:** 10.1186/1880-6805-31-10

**Published:** 2012-05-04

**Authors:** Tomoko Morinaka, Malgorzata Wozniewicz, Jan Jeszka, Joanna Bajerska, Porn-ngarm Limtrakul, Luksana Makonkawkeyoon, Naoko Hirota, Shoko Kumagai, Yoshiaki Sone

**Affiliations:** 1 Graduate School of Human Life Science, Osaka City University, Sugimoto, Sumiyoshi-ku, Osaka, Japan; 2 Department of Human Nutrition and Hygiene, University of Life Sciences in Poznan, Poznan, Poland; 3 Department of Biochemistry, Faculty of Medicine, Chiang Mai University, Chiang Mai, Thailand; 4 Faculty of Human Health Science, Matsumoto University, Matsumoto, Japan

**Keywords:** Body composition, Macronutrient intake, Overnight fast, Percent body fat, Respiratory quotient (RQ), Seasonal variation

## Abstract

**Background:**

From the viewpoint of human physiological adaptability, we previously investigated seasonal variation in the amount of unabsorbed dietary carbohydrates from the intestine after breakfast in Japanese, Polish and Thai participants. In this investigation we found that there were significant seasonal variations in the amount of unabsorbed dietary carbohydrates in Japanese and Polish participants, while we could not find significant seasonal variation in Thai participants. These facts prompted us to examine seasonal variations in the respiratory quotient after an overnight fast (an indicator of the ratio of carbohydrate and fat oxidized after the last meal) with female university students living in Osaka (Japan), Poznan (Poland) and Chiang Mai (Thailand).

**Methods:**

We enrolled 30, 33 and 32 paid participants in Japan, Poland and Thailand, respectively, and measurements were taken over the course of one full year. Fasting respiratory quotient was measured with the participants in their postabsorptive state (after 12 hours or more fasting before respiratory quotient measurement). Respiratory quotient measurements were carried out by means of indirect calorimetry using the mixing chamber method. The percent body fat was measured using an electric bioelectrical impedance analysis scale. Food intake of the participants in Osaka and Poznan were carried out by the Food Frequency Questionnaire method.

**Results:**

There were different seasonal variations in the fasting respiratory quotient values in the three different populations; with a significant seasonal variation in the fasting respiratory quotient values in Japanese participants, while those in Polish and Thai participants were non-significant. We found that there were significant seasonal changes in the percent body fat in the three populations but we could not find any significant correlation between the fasting respiratory quotient values and the percent body fat.

**Conclusions:**

There were different seasonal variations in the fasting respiratory quotient values in the three different populations. There were significant seasonal changes in the percent body fat in the three populations but no significant correlation between the fasting respiratory quotient values and the percent body fat.

## Background

There is a Japanese saying, ‘the autumn climate is so good that even horses get fat’. This means that the autumn harvest season, especially for the Japanese staple food (rice), is the time when people stockpile food in order to survive severe winters when food is less available. This is also the time horses are fed hay to fatten them. From a human evolutionary viewpoint, it is generally accepted that storage of energy-rich fats is more efficient in periods of food abundance so as to survive food shortages. The function of this fat storage mechanism is to have a source of energy available for times when food is scarce or difficult to find, or when animals hibernate [1]. It is well-known that hibernating mammals are able to store large amounts of energy-rich lipids before hibernation, and part of the mechanism is assumed to be accomplished by an increase in adipose tissue lipoprotein lipase [2]. From the viewpoint of human nutritional evolution, it is very interesting to see that modern populations still have a physiological mechanism to control the seasonal change in fat accumulation as hibernant animals do, even though modern populations are surrounded by readily available energy-rich food throughout the year more so than ever before.

Concerning seasonal variations in the percent body fat of Japanese university students, Yamashita *et al*. [3] surveyed the seasonal variations in the percent body fat among young Japanese university students based on sex and regional differences. They found that there is a significant seasonal change in the percent body fat among female university students living in metropolitan areas, where it is higher in winter and lower in summer. In addition, they concluded that only the seasonal factor significantly contributed to changes in the percent body fat [4]. These reports suggest that modern young Japanese people still have an intrinsic physiological mechanism to control body fat storage according to seasonal changes in their living environment.

During the course of our investigation on the effects of the environment on the human digestive system, we measured seasonal variations in the amount of unabsorbed dietary carbohydrates (hereafter the amount of unabsorbed dietary carbohydrates is abbreviated as AUDC) from the intestine after breakfast in Japanese female university students (in 2003 to 2004) and Polish participants (in 2004 to 2005). In these surveys, we found that the AUDC from the intestine was larger in winter and smaller in autumn than in any other season in Japanese and Polish participants [5,6]. These results indicate that carbohydrate absorption in the intestine is better in autumn than in winter. This is consistent with the seasonality of insulin secretion reported by Haus *et al*. [7] with normal participants living in Minnesota (located in central-north USA), where insulin responses to the three meals were in the following order: autumn > summer > spring > winter. To determine the regional difference in seasonality in the efficiency of dietary carbohydrate absorption from the intestine, we examined seasonal variations in the AUCD in Chiang Mai, tropical Thailand, using the same method as that for the Japanese and Polish participants. In contrast to the results obtained with Japanese and Polish participants, we could not find any significant seasonal variation in the AUCD of young female Thai university students [8]. These results obtained with Japanese, Polish and Thai participants indicate that there are different seasonally changing patterns in the efficiency of dietary carbohydrate absorption in the intestine in the different populations.

Considering the human metabolism, we can speculate that seasonal change in the efficiency of carbohydrate absorption in the intestine may have a relationship with the balance of carbohydrate and fat metabolism. This is probably through seasonal changes in insulin response to the diets described by Haus *et al*. [7], because insulin is a key hormone in the regulation of carbohydrate and fat metabolism in many organs and tissues in our body. In addition, it is generally accepted that an increase in the percent body fat results from triacylglycerol accumulation in adipose tissue over a long period (months or years), and that whole body integration of carbohydrate and fat metabolism plays an important role in the regulation of the fat accumulation process [9]. Thus we can postulate that people living in different regions have different seasonally changing patterns in the balance of carbohydrate and lipid metabolism, and it correlates to the seasonal changes in the percent body fat.

We measured the participants’ respiratory quotient (RQ) in everyday life after an overnight fast (an indicator of the ratio of carbohydrate and fat being oxidized in the participants’ postabsorptive state, before breakfast in the morning) to find seasonal variation in the balance of the carbohydrate and fat metabolism. The participants’ percent body fat and body weight were measured to determine seasonal changes in body fat accumulation. We also measured their food intake to determine seasonal change in macronutrient intake. We carried out this series of examinations in three metropolitan cities located in different climate zones, Osaka (Japan), Poznan (Poland) and Chiang Mai (Thailand), in the same calendar months, April to May, July to August, October to November (2008), and January to February (2009) in Japan, and 2009 to 2010 in Poland and Thailand. Monthly mean temperature and precipitation in the year of the RQ examination and the average in the years of 1981 to 2010 are summarized in Table 1.

**Table 1:** Monthly mean temperature and precipitation in Osaka, Poznan and Chiang Mai

	**Apr**	**May**	**Jul**	**Aug**	**Oct**	**Nov**	**Jan**	**Feb**
**Osaka, Japan**								
Temperature (°C)	15.4(15.1)	20.0(19.7)	28.7(27.4)	28.4(28.8)	19.6(19.0)	13.4(13.6)	6.5(6.0)	7.9(6.3)
Precipitation (mm)	144.0(103.8)	219.0(145.5)	124.0(157.0)	82.0(90.9)	62.0(112.3)	50.0(69.3)	73.0(45.4)	95.0(61.7)
**Poznan, Poland**								
Temperature (°C)	12.1(8.7)	13.6(13.9)	19.5(18.9)	19.6(18.3)	7.5(8.9)	6.6(3.8)	−6.5(−0.9)	−1.0(0.0)
Precipitation (mm)	20.0(31.6)	86.0(48.1)	86.0(74.3)	24.0(58.5)	52.0(32.4)	35.0(34.6)	28.0(33.3)	18.0(28.4)
**Chiang Mai, Thailand**								
Temperature (°C)	29.6(29.3)	28.5(28.7)	27.8(27.5)	28.0(27.3)	27.4(26.5)	25.1(24.3)	24.2(22.0)	24.5(24.0)
Precipitation (mm)	98.0(57.9)	142.0(158.8)	124.0(135.4)	126.0(208.4)	223.0(117.6)	0.0(58.5)	22.0(4.1)	0.0(9.5)

The figures shows monthly mean temperature and precipitation in the year of the respiratory quotient examination and those in parenthesis are the average of 1981 to 2010. These data were obtained from the metrological information of Japan Meteorological Agency (http://www.data.jma.go.jp/gmd/cpd/climatview_jp/index.html[30]).

In this paper, we describe seasonal variations in the fasting RQ values, the percent body fat, body weight and the macronutrient intake in the different populations, and discuss the relationship between them.

## Methods

### Participants

Because some people have irregular breathing (for example, hyperpnoea), we first examined the RQ of 40 (in Japan), 44 (in Poland) and 39 (in Thailand) healthy female university students in April and May, at the start of a one year examination (2008 in Japan, 2009 in Poznan and Chiang Mai). Based on the participants’ breathing stability judged by the monitoring of expiratory minute volume (V_E_) on the metabolic analyzer, we selected 30, 33 and 32 students for measurement over the course of one full year as paid participants in Japan, Poland and Thailand, respectively. All participants in the three countries were non-smokers and healthy based on self-definition with no history of metabolic disorders. The participants were female university students belonging to the Universities of the authors (Department of Foods and Nutrition, Osaka City University in Japan; Department of Human Nutrition and Hygiene, University of Life Sciences in Poznan in Poland; and Faculty of Medicine, Chiang Mai University in Thailand). The number of participants who completed participation in all four seasons (some were forced to retire from the examination after participation in April and May due to personal reasons or disease) was 30 in Japan, 32 in Poland) and 30 in Thailand. From among them we excluded six, two, and three participants respectively from further data analysis due to observation of RQ values over 1.00 or under 0.70 (normal range: 1.00 to 0.70) in one season’s examination. Table 2 shows participants’ mean age, height (recollected value in Thailand), body weight, body mass index and percent body fat of the participants whose RQ and other data were subjected to further analysis (24 Japanese, 30 in Polish and 27 Thai participants).

**Table 2:** Characteristics of the participants

	**Japanese (*n*= 24)**	**Polish (*n*= 30)**	**Thai (*n*= 27)**
Age (years)	18.3 ± 0.6(18 to 20)	21.0 ± 1.4(19 to 23)	22.9 ± 2.7(18 to 27)
Height (cm)	160.0 ± 5.6(152.8 to 174.5)	168.9 ± 6.8(150.0 to 181.5)	159.1 ± 5.3(149.0 to 170.0)
Weight (kg)	49.8 ± 5.5(39.6 to 58.6)	58.2 ± 7.7(46.3 to 74.6)	46.7 ± 5.6(38.9 to 60.3)
Body mass index (kg/m^2^)	19.5 ± 2.0(16.8 to 23.7)	20.4 ± 2.2(17.3 to 25.2)	18.4 ± 2.0(15.7 to 22.8)
Body fat (%)	22.5 ± 3.9(13.1 to 29.7)	24.4 ± 3.9(17.0 to 36.0)	22.9 ± 3.7(16.6 to 29.8)

The values are mean, standard deviation, and range of characteristics. These values were obtained when the examination started, Apr to May 2008 for Japan and on Apr to May 2009 for Poland and Thailand.

Participants underwent the RQ measurement in the follicular or luteal phase of their menstrual cycles (not during their menstrual period) because Paolisso *et al*. [10] reported that no significant difference was found in the fasting RQ values throughout the different phases of the menstrual cycle. In addition, we also confirmed in our preliminary examination, using Japanese female students belonging to the same department as that of the participants participating in this study, that there was no significant difference in the fasting RQ values between those in the follicular phase and the luteal phase.

We explained the purpose of the study and the procedures involved to all the participants before they gave their written consent to participate. This was done according to the protocol approved by the Research Ethics Committee of the Graduate School of Human Life Science, Osaka City University (approval No. 08/01), Poznan Medical Ethics Committee, Poznan University of Medical Sciences (approval No. 344/2009), and the Research Ethics Committee 3 of the Faculty of Medicine, Chiang Mai University (approval No. 074/2009). These three approvals are based on the principles of the Declaration of Helsinki of the World Medical Association and the conduct of the research adhered to the ethical principles and standards of chronobiological study [11].

### Experiment protocol and measurements

**Figure 1:**
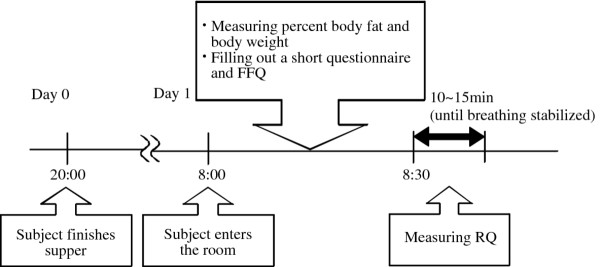
**Experimental protocol.** The protocol was the same in the four seasons and in the three populations.

In this study, we used the same experimental protocol and the same model of measuring instruments, including indirect calorie calorimetry and electric bioelectrical impedance analysis scale, in Japan, Poland and Thailand. Figure 1 shows the experiment protocol. Each participant was requested to have her evening meal by 10 p.m. on the day before participation in the RQ examination. We gave no suggestions on the menu for the meal, but strongly requested that they have no snacks or caffeinated beverages between their evening meal and completion of the examination on the morning of the following day.

Usually, three participants were examined in a morning: the first entered the room at 8 a.m., the second at 8.20 a.m. and the third at 8.40 a.m. The participants remained sedentary in an air-conditioned examination room under normal fluorescent lamps (300 to 400 lux) until the end of the experiment. Approximate average room temperatures of the examination rooms were controlled to around 24°C to 26°C in Japan, 21°C to 22°C in Poland and 24°C to 25°C in Thailand.

For the first 30 min after entering the room, participants were requested to allow measurement of their percent body fat (including body weight) without their outer wear and to fill out a short questionnaire concerning, for example, hours of sleep and time of their last evening meal. Each participant was also asked to fill out the Food Frequency Questionnaire (FFQ) for the previous month. During this 30 min period between entering and measurement of RQ, participants’ breathing returned to normal and they acclimatized to the atmosphere of the room. Body weight was measured to the nearest 0.1 kg using an Omron HBF-352-W electric scale (Omron Corporation, Kyoto, Japan). In this study, participants were weighed in their usual clothing without their outer wear, and a correction of 1.0 kg was made to account for the weight of this clothing. The percent body fat was measured using an Omron HBF-352-W electric bioelectrical impedance analysis scale.

Fasting RQ was measured with the participants in their postabsorptive state (after 12 hours or more fasting before RQ measurement) in a sedentary position. RQ measurements were carried out by means of indirect calorimetry using the mixing chamber method with a Portable Gas Monitor AR-1 (type 4, Arco System, Chiba, Japan) connected to a facemask. The gas analyzer in this system is equipped with a galvanic battery type oxygen sensor and infrared absorption type carbon dioxide sensor. It was calibrated through two steps: air calibration and precise calibration using a standard gas containing 4.95 % carbon dioxide balanced with nitrogen. In this system, calculated V_E_ (L/min), oxygen uptake (mL/min), carbon dioxide production (mL/min) and RQ (at one minute intervals) were shown in real time in the display area. We measured the RQ for 15 min while the participant’s breathing was stable by monitoring of RQ on the display. Therefore, RQ values are expressed as mean RQ values per minute. Careful maintenance of this machine guarantees accuracy of RQ value to two decimal places.

### Food intake survey

In order to evaluate average food intake (in this paper we described carbohydrate, fat and protein intakes) of the young female university students in Osaka, Japan and Poznan, Poland, we applied the FFQ method, but the exact questionnaires used in each country were different because we had to refer to the Food Composition Table of each country in order to calculate nutrient intake. We asked healthy female university students, including the participants, for the RQ measurement (100 Japanese participants and 111 Polish participants) to answer the questionnaire. We asked the students to answer questions about their diet history for one month before the day they answered the questions. In the Japanese center, we applied the Diet History Questionnaire system, where the participant’s nutritional intake was calculated using an *ad hoc* computer program (DHQBox system 2008, EBNJapan, Tokyo, Japan) developed by Sasaki *et al*. [12]. In the Polish arm, the food intake survey was conducted using an FFQ prepared for this project based on its brief version as described by Wozniewicz *et al*. [13]. (In Thailand, because a validated FFQ method was not available for Chiang Mai when we started RQ measurement in 2009, we prepared a FFQ citing 121 northern Thai foods. This FFQ has not been validated by other food intake survey methods yet, therefore we did not present food intake data of Thai participants in this paper.)

The protein, fat, carbohydrate and alcohol intakes were calculated as energy-adjusted nutrient intakes, which indicate the nutrient composition of the diet by regressing nutrient intakes on total energy [14]. The ratio of carbohydrate to fat intake (C/F ratio) was calculated by dividing the percentage of carbohydrate intake by the percentage of fat intake (see Table 3). In this paper, we only show the results of the food intake survey of the participants who participated in the RQ and the percent body fat measurements. A further detailed analysis of the food intake survey of over 100 participants, including the participants for the RQ measurement, will be presented in another paper.

**Table 3:** Summary of nutrients intake and carbohydrate to fat intake ratio of Japanese and Polish participants

**Month**	**Energy intake (kcal)**	**Protein (%)**	**Fat (%)**	**Carbohydrate (%)**	**C/F ratio**
**Japanese (n = 24)**					
Apr to May	1685.2 ± 429.5	14.1 ± 2.0	29.0 ± 7.4	56.4 ± 7.6	2.12 ± 0.79
Jul to Aug	1745.1 ± 441.1	13.9 ± 2.0	28.8 ± 4.3	56.7 ± 4.7	2.03 ± 0.45
Oct to Nov	1716.3 ± 479.4	13.8 ± 1.5	27.4 ± 5.0	58.6 ± 4.9	2.24 ± 0.62
Jan to Feb	1608.0 ± 493.2	14.6 ± 2.0	28.1 ± 5.1	56.6 ± 5.8	2.11 ± 0.57
**Polish (n*=*30)**					
Apr to May	1858.5 ± 406.8	16.2 ± 2.3	33.5 ± 5.5	48.9 ± 6.0^a^	1.53 ± 0.42^a^
Jul to Aug	1856.7 ± 674.4	15.4 ± 2.2	30.7 ± 3.8	52.7 ± 4.1^a^	1.76 ± 0.34^a^
Oct to Nov	1846.3 ± 576.4	14.8 ± 2.5	32.6 ± 6.3	51.0 ± 7.7	1.68 ± 0.66
Jan to Feb	1742.1 ± 561.0	15.1 ± 2.2	33.1 ± 5.3	50.2 ± 6.2	1.58 ± 0.42

The C/F ratio is expressed by as the percentage of energy from carbohydrate intake divided by the percentage of energy from fat intake. These values are those of Japanese and Polish participants who participated in RQ and body fat examination. ^a^Means sharing a common superscript letter are significantly different, *P* <0.05. C/F ratio: carbohydrate to fat intake ratio.

### Data analysis

All data were expressed in terms of means ± SD. We analyzed seasonal variations in the fasting RQ values, the percent body fat, body weight and nutrient intake (in Japan and Poland) by one-factor (season) repeated measure analysis of variance followed by post hoc multiple comparison tests using the Bonferroni method in each country. The correlation between the variables such as the fasting RQ value, the percent body fat, energy intake, fat intake, carbohydrate intake and C/F ratio within the participant was analyzed by multiple regression with repeated observations [15]. A *P* <0.05 was considered to be statistically significant.

## Results

As described in the Participants section, we analyzed seasonality of the fasting RQ values, the percent body fat and body weight of the 24, 30 and 27 participants (in Japan, Poland and Thailand, respectively) who could participate in all four periods. Table 4 summarizes the mean and standard deviations of the fasting RQ values, the percent body fat and body weight (measured weight minus 1 kg of clothes) of the participants in the three populations and in the four seasons with indication of statistical differences. Interactions of the two factors, country and season, in the seasonal change in the fasting RQ values was shown graphically and two-factor (country and season) analysis of variance showed a borderline value, (*F* (6, 234) = 1.989, *P* = 0.068). Therefore, we analyzed the seasonal variation of the variables described above by one-factor (season) repeated measure analysis of variance followed by post hoc multiple comparison tests using the Bonferroni method in each country.

**Table 4:** Summary of the mean and standard deviations of the measurements

**Month**	**Fasting respiratory quotient**	**Percent body fat (%)**	**Weight (kg)**
**Japanese (n = 24)**			
Apr to May	0.812 ± 0.034^a^	22.5 ± 3.9^a^	49.8 ± 5.5
Jul to Aug	0.802 ± 0.055^b^	20.7 ± 4.4^abc^	49.1 ± 5.1
Oct to Nov	0.833 ± 0.040	23.3 ± 5.1^b^	49.4 ± 5.7
Jan to Feb	0.838 ± 0.045^ab^	23.5 ± 4.3^c^	50.1 ± 6.3
**Polish (n = 30)**			
Apr to May	0.816 ± 0.035	24.4 ± 3.9	58.2 ± 7.7
Jul to Aug	0.811 ± 0.043	23.9 ± 3.8^ab^	58.0 ± 7.7^a^
Oct to Nov	0.816 ± 0.040	25.3 ± 3.8^a^	59.1 ± 8.3^a^
Jan to Feb	0.823 ± 0.035	25.1 ± 3.8^b^	58.3 ± 7.9
**Thai (n = 27)**			
Apr to May	0.807 ± 0.055	22.9 ± 3.7	46.7 ± 5.6
Jul to Aug	0.817 ± 0.058	23.0 ± 3.7^a^	46.3 ± 5.6
Oct to Nov	0.811 ± 0.040	23.8 ± 3.3^a^	46.8 ± 5.8
Jan to Feb	0.807 ± 0.035	23.8 ± 3.6	46.7 ± 5.4

Participants were weighed in their usual clothing without their outer wear, and a correction of 1.0 kg was made to account for the weight of this clothing. ^a,b,c^Means sharing a common superscript letter are significantly different, *P* <0.05.

### Fasting respiratory quotient

**Figure 2:**
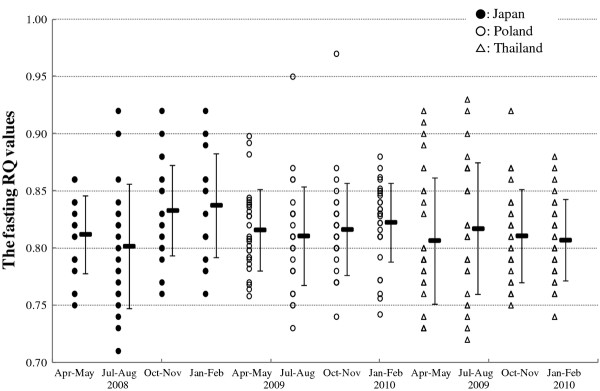
**Seasonal variation in fasting respiratory quotient values of Japanese, Polish and Thai participants.** Broad and narrow bar show mean value and SD, respectively. Closed circle: Japanese; open circle: Polish; triangle: Thai.

Figure 2 depicts seasonal variation in the fasting RQ values of Japanese, Polish and Thai participants. One-factor (season) repeated measures analysis of the variance of the RQ values in the four seasons (Apr to May of 2008 to Jan to Feb of 2009) of Japanese participants showed that there was a significant seasonal variation in the fasting RQ values, (*F* (3, 69) = 4.957, *P* = 0.004). Post hoc multiple comparison tests indicated that the RQ values in Apr to May and Jul to Aug were significantly lower than in Jan to Feb (*P* = 0.043 and 0.033, respectively). The RQ value in Apr to May was not significantly different from those in Jul to Aug and Oct to Nov (*P* = 1.000 and *P* = 0.240, respectively). The RQ value in Oct to Nov was not significantly different from those in Jul to Aug and Jan to Feb (*P* = 0.193 and *P* = 1.000, respectively). In Polish participants, there was no significant seasonal variation in the fasting RQ values, (*F* (3, 87) = 0.666, *P* = 0.575). In addition, there was no significant seasonal variation in the fasting RQ values, (*F* (3, 78) = 0.331, *P* = 0.803) in Thai participants.

### Percent body fat and body weight

**Figure 3:**
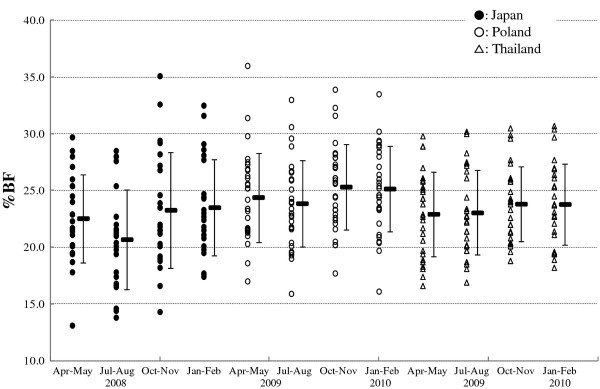
**Seasonal variation in the percent body fat of Japanese, Polish and Thai participants.** Broad and narrow bar show mean value and SD, respectively. Closed circle: Japanese; open circle: Polish; triangle: Thai.

Figure 3 shows seasonal variation in the percent body fat of Japanese, Polish and Thai participants. One-factor (season) repeated measures analysis of the variance of the percent body fat of Japanese participants showed that there was a significant seasonal variation in the percent body fat, (*F* (3, 69) = 5.787, *P* = 0.001). In addition, the post hoc multiple comparison indicated that the percent body fat in Jul to Aug was significantly lower than those in Apr to May (*P* = 0.002), Oct to Nov (*P* = 0.033) and Jan to Feb (*P* = 0.003). In Polish participants, there was a significant seasonal variation in the percent body fat, (*F* (3, 87) = 9.332, *P* < 0.0005). The post hoc multiple comparison indicated the percent body fat in Jul to Aug was significantly lower than those in Oct to Nov (*P* <0.0005) and Jan to Feb (*P* = 0.008). In Thai participants, there was a significant seasonal variation in the percent body fat, (*F* (3, 78) = 4.427, *P* = 0.006). Post hoc multiple comparison indicated that percent body fat in Jul to Aug was significantly lower than that in Oct to Nov (*P* = 0.042).

In Table 4, we summarize seasonal variation in the body weight of Japanese, Polish and Thai participants. One-factor (season) repeated measures analysis of the variance of body weight of Japanese participants indicated that there was no significant seasonal change (*F* (3, 69) = 2.029, *P* = 0.118). In Polish participants, there was a significant seasonal variation in body weight, (*F* (3, 87) = 4.072, *P* = 0.009). The post hoc multiple comparisons showed body weight in Jul to Aug was significantly lower than that in Oct to Nov (*P* = 0.010). In Thai participants, there was no significant seasonal variation in body weight, (*F* (3, 78) = 1.193, *P* = 0.318).

### Energy, macronutrient intake and carbohydrate to fat intake ratio

Energy, protein, fat and carbohydrate intakes and C/F ratios of Japanese and Polish young female university students who participated in the RQ measurement are summarized in Table 3. As the FFQ analyzing systems that we used in Japan and Poland have been verified by a direct food intake survey [12,13] , we subjected food intake values of each participant to the statistical analysis to determine their seasonality.

In Japanese participants, there was no seasonal variation in any variables: total energy intake (*F* (3, 69) = 0.838, *P* = 0.478), protein intake (*F* (3, 69) = 1.753, *P* = 0.164), fat intake (*F* (3, 69) = 0.686, *P* = 0.564), carbohydrate intake (*F* (3, 69) = 1.167, *P* = 0.329), or C/F ratio (*F* (3, 69) = 0.786, *P* = 0.506).

In Polish participants, there was no significant seasonal variation in total energy intake (*F* (3, 87) = 0.636, *P* = 0.594). Protein intake ((*F* (3, 87) = 4.186, *P* = 0.008) and fat intake (*F* (3, 87) = 2.887, *P* = 0.040) demonstrated significant seasonal changes but a post hoc multiple comparison test showed no significantly difference among the seasons. As for the seasonality of the C/F ratio, there was no significant variation (*F* (3, 87) = 2.659, *P* = 0.053) while a post hoc multiple comparison test showed there was significant difference between that of Apr to May and that of Jul to Aug (*P* = 0.035). There was a significant seasonal change in carbohydrate intake (*F* (3, 87) = 3.667, *P* = 0.015), where the carbohydrate intake in Apr to May was significantly lower than that in Jul to Aug (*P* = 0.019) with a post hoc multiple comparison test.

### Correlation between the variables measured in this study

We calculated correlation coefficients within participants between the fasting RQ values and the percent body fat, and those with the variables of the food intake survey in this study, by multiple regression with repeated observations. Table 5 shows the results of these analyses and indicated that there were no significant correlations within participants between any pairs of variables.

**Table 5:** Bivariate correlation of respiratory quotient, percent body fat and nutrient intake

**Independent variable**	**Dependent variable**	**Regression slope**	**Correlation coefficient within participants**	***P***
**Japanese (n = 24)**				
Energy intake (kcal)	RQ	0.000	0.062	0.604
Fat (%)	RQ	0.000	0.036	0.763
Carbohydrate (%)	RQ	0.000	0.025	0.834
C/F ratio	RQ	0.005	0.058	0.625
RQ	%BF	13.360	0.189	0.108
Energy intake (kcal)	%BF	−0.001	−0.119	0.314
Fat (%)	%BF	0.009	0.014	0.908
Carbohydrate (%)	%BF	−0.048	−0.076	0.522
C/F ratio	%BF	−0.162	−0.027	0.820
**Polish (n = 30)**				
Energy intake (kcal)	RQ	0.000	0.131	0.217
Fat (%)	RQ	−0.001	−0.088	0.409
Carbohydrate (%)	RQ	0.001	0.112	0.291
C/F ratio	RQ	0.009	0.102	0.335
RQ	%BF	8.349	0.192	0.068
Energy intake (kcal)	%BF	0.000	0.066	0.533
Fat (%)	%BF	0.063	0.188	0.075
Carbohydrate (%)	%BF	−0.040	−0.136	0.198
C/F ratio	%BF	−0.697	−0.179	0.090
**Thai (n = 27)**				
RQ	%BF	−4.629	−0.159	0.154

C/F ratio: carbohydrate to fat intake ratio; %BF: percent body fat; RQ: respiratory quotient. Correlation between the variables within the subject was analyzed by multiple regression with repeated observations. *P* <0.05 was considered to be statistically significant.

## Discussion

Before discussion of the present results, we will describe the meteorological characteristics of the three cities, Osaka, Poznan and Chiang Mai, and the definition of fasting RQ from the point of carbohydrate and fat metabolism.

Table 1 shows the monthly mean temperature and precipitation in the years of 1981 to 2010 (the average) and the year of the RQ examination. On average, we can categorize each two-month period according to the order of mean temperatures as follows: in Osaka (Japan) and Poznan (Poland); Jul to Aug (the hottest season, summer), Oct to Nov (autumn), Apr to May (spring) and Jan to Feb (the coldest season, winter). In Chiang Mai, they were categorized according to monthly mean temperatures and precipitation: Apr to May (hot season), Jul to Aug (rainy season), Oct to Nov (dry season), and Jan to Feb (dry and cool season). According to the World map of Koppen-Geiger climate classification [16], Osaka is located in a warm oceanic climate zone, Poznan is in the continental climate zone, and Chiang Mai is in a tropical monsoon climate zone.

The definition of the fasting RQ value is the RQ value in the postabsorptive state, where the postabsorptive state is typically represented by the state after an overnight fast before breakfast is consumed. In this phase, all of the meal has been absorbed from the intestinal tract [9]. In this study, participants came to the experimental room from their residences by their usual means of transportation having performed their usual physical activities before the RQ examination, but having fasted for 12 hours or more. Their RQ values were therefore classed as fasting RQ values in everyday life. In a postabsorptive state, the concentrations of glucose and insulin are at their lowest and the concentration of non-esterified fatty acids is at its highest in a day [17]. In this study, we neglected the contribution of protein oxidation to total RQ values by not measuring urea excretion because nutrients oxidized in well-nourished people are mostly carbohydrates and lipids [18]. In addition, it is important to note that, in this postabsorptive state, glucose that enters the blood is almost exclusively from the liver (a proportion arises from glycogen breakdown and a proportion from gluconeogenesis) and non-esterified fatty acids are released by the action of hormone-sensitive lipase on the triacylglycerol stores in adipose tissue. Therefore, the nutritional composition of food ingested in the previous day or the day before (in a short-term) does not directly affect the fasting RQ values.

### Seasonal variation in the fasting respiratory quotient values

There was a significant seasonal variation in the participants in Osaka, and the mean RQ value in Jul to Aug was significantly lower than that in Jan to Feb (the lowest mean value in the four seasons); those values were in the order of Jan to Feb > Oct to Nov > Apr to May > Jul to Aug. Table 5 shows the bivariate correlation of the fasting RQ and four items of food intake survey, indicating no correlation between them in Japanese participants. This lack of correlation between the RQ values and the items of food intake suggests that the nutritional composition of foods ingested had no effect on the fasting RQ values in the long-term in Japanese participants. In addition, there were no seasonal change in energy, fat or carbohydrate intake, as well as C/F ratio (see Table 3). These facts indicate that there are some factors other than food intake affecting the seasonal variation in the fasting RQ values.

Using the RQ values obtained in this study, we can calculate and estimate the ratio of fat oxidation to the total oxidation of carbohydrate and lipids by the following equation: (1.00-RQ)/(1.00-0.70). Application of this equation gives the ratios of carbohydrates metabolized to lipids metabolized in the four seasons in the participants in Osaka as follows: Jan to Feb (0.46: 0.54), Oct to Nov (0.44: 0.56), Apr to May (0.37: 0.63) and Jul to Aug (0.34: 0.66). These ratios indicate that relatively more lipids were metabolized in Jul to Aug (summer) and Apr to May (spring) compared to in Oct to Nov (autumn) and Jan to Feb (winter). These results agree with several reports about human seasonal variation in fasting serum glucose and triglyceride levels. For example, Behall *et al*. [19] observed that fasting serum glucose level was in the order of Dec to Feb > Sep to Nov > Mar to May > Jun to Aug in in young women living in California, USA and Gordon *et al*. [20] reported that there was a significant fall in triglyceride level from summer to winter in participants living in London, UK. This seasonality reported in the fasting serum glucose and lipid levels is consistent with the seasonal variation in the fasting RQ values observed in this study; in winter, the more fasting serum glucose there is, the more glucose is metabolized and the higher the obtained fasting RQ values are. In contrast, in summer, the more fasting triglyceride there is, the more fat is metabolized, the lower the obtained RQ values are. There are, however, few reports about seasonality in fasting serum glucose and plasma triglyceride levels of modern Japanese young female participants. In order to make it clear that our postulation described above is one of the possible mechanisms of the seasonal variation in the fasting RQ values observed in Japanese participants, we need further examination of seasonal variation in fasting serum glucose and triglyceride levels of the Japanese population, and seasonal change in longer-term control of gene expressions related to carbohydrate and lipid metabolism.

In the participants of Poznan, as described in the report of Tsumura *et al*. [6], there was significant seasonal variation in the efficiency of carbohydrate absorption in the intestine similar to that of Japanese participants; however, there was no significant seasonal variation in the fasting RQ values in this study. Our speculation, described in the Background, is that seasonal change in the efficiency of carbohydrate absorption in the intestine may have a relationship with the balance of carbohydrate and fat metabolism as observed in Japanese participants. Comparison of the fasting RQ value between Japanese and Polish participants shows that the mean RQ value of Polish participants in Jul to Aug was relatively higher than that of the Japanese participants. These higher RQ values in Jul to Aug might result in a non-significant seasonal variation in the fasting RQ values. One possible reason for the observed relatively higher RQ values in the summer period might be related to the significantly higher carbohydrate intake during Jul to Aug periods (see Table 3), on the assumption that this affects the carbohydrate and fat metabolism accompanied by other seasonal factors, such as changes in temperature and/or light intensity, which are peculiar to Polish living environments. Here, we can refer to studies by Plasqui *et al*. [21] and Oshiba [22], who reported that seasonal environmental changes affect total thyroxine or thyroid-stimulating hormone levels that influence human metabolic rate (probably the carbohydrate/fat metabolism). Of course we need to have more evidence concerning this assumption.

In Thai participants, there was no seasonal variation in the fasting RQ values. This could be explained by our previous finding that there was no significant seasonal variation in the AUCD of young female Thai university students [8].

### Seasonal variation in the percent body fat and its relation to the seasonality in the fasting respiratory quotient values

In this study, we analyzed the percent body fat measured by an electric bioelectrical impedance analysis scale to provide an estimation of the relationship between the seasonality of fasting RQ values and that of the percent body fat in each population. The lack of a simple and international standard method for evaluation of exact body composition constituted a limitation to our study.

In Japanese participants, the seasonality of the percent body fat decreased in the order Jan to Feb > Oct to Nov > Apr to May > Jul to Aug. The percent body fat in the period of Jul to Aug was significantly lower than that in the other three periods. As described in the Background, Yamashita *et al*. [3] found that there was a significant seasonal change in the percent body fat among female university students living in metropolitan areas, where it is highest in winter and lowest in summer, which is consistent with the results obtained in this study. In addition, a similar seasonality of body fat percentage was reported by Mori *et al*. [23]. They investigated seasonal change of fat mass in thirteen women of 20 to 30 years old using magnetic resonance imaging and reported that body weight, percent body fat, the subcutaneous fat mass and subcutaneous fat thickness of the abdomen were highest in January and lowest in July. These previous reports and our present result suggest that modern young Japanese females still have some regulatory mechanism of fat accumulation according to seasonal changes in their circumstances. This mode of seasonal changing in the percent body fat, decreasing in spring to summer and increasing in autumn to winter, was similar to that of the fasting RQ values in Japanese participants.

This similarity in seasonal changes between the fasting RQ values and the percent body fat observed in Japanese participants may represent a possible physiological mechanism. We postulate that Japanese participants may metabolize more fat to lose fat storage in the hot season, but metabolize more carbohydrate to save fat for storage in the colder season under an almost constant energy intake and expenditure, as described in the Background section. This constancy was shown in Table 3, which demonstrated that there was no significant seasonal variation in the energy intake of Japanese participants, and by our previous study, which showed there was no significant seasonal variation in the normal daily physical activity of Japanese university female students, as measured by a small accelerometer [5]. Our hypothesis might be supported by a report concerning the seasonal variation in adipose tissue lipoprotein lipase, which provides free fatty acids for storage in adipocytes; where it was higher in winter than in summer as observed in participants in Colorado, USA [24]. In order to examine this postulation, we calculated within-participant correlation coefficients between the fasting RQ values and the percent body fat, on the assumption that the correlation will be significant if our postulation is one of the possible regulatory mechanisms. However, Table 5 shows that there was no significant correlation within participants between the fasting RQ values (as the independent variable) and the percent body fat (dependent variable) in Japanese participants. This result indicates that seasonal variation in the balance of carbohydrate and fat metabolism has no significant effect on the seasonality in the percent body fat.

In Polish participants, there was a significant seasonal variation in the percent body fat; the order was that of Oct to Nov > Jan to Feb > Apr to May > Jul to Aug. This mode of changing was very similar to that of Japanese participants, where the percent body fat was the lowest in Jul to Aug and relatively higher in Oct to Nov and Jan to Feb. To examine the correlation between the seasonality in the fasting RQ values and the percent body fat in Polish participants, we calculated correlation coefficient values within participants and found that there was no significant correlation between them (Table 5). This result, together with that of Japanese participants, contradicts our postulation. In this correlation analyses, however, the correlation coefficient values of Japanese and Polish participants were nearly close to 0.200 (which is generally accepted as the least value suggesting very weak correlation). The borderline probability in both participant groups may allow us to speculate that there was a big seasonal effect on the balance of carbohydrate and fat metabolism, and so on fat accumulation, in our ancestors long ago, but the effect has become so small that it no longer has any influence on our percentage of body fat, particularly because our ancestors spent their daily life in a much more food-scarce and less artificial environment than the modern human population. Modern human beings, especially those living in cosmopolitan areas, are surrounded by artificial environments, such as the constant temperature provided by air-conditioning and foods available at any time. This speculative theory is supported by other examples of the loss of seasonality in our metabolic physiology: Nakamura *et al*. [25] reported that seasonal variations in basal metabolic rate were getting small, especially in indoor sedentary workers; and Maeda *et al*. [26] reported that there was no seasonal variation in the basal metabolic rate in university students. These authors considered that the improvement of air-conditioning influences the artificial microclimate and might reduce the seasonal variation in the basal metabolic rate of the Japanese population.

One more possible factor that affected the present correlation analysis is the participants’ degree of obesity (or leanness). There is a possibility that the serum non-esterified fatty acid level relates to the amount of fat storage in the adipose tissue, that is, the degree of obesity. We should have analyzed within-participant correlation coefficients between the fasting RQ values and the percent body fat, but we could not analyze the correlation statistically under the stratification of the degree of obesity due to the small number of the participants participated in this study.

In Thai participants, there was also a significant seasonal change in the percent body fat; the order was that of Oct to Nov > Jan to Feb > Jul to Aug > Apr to May. It is noteworthy that there was a common feature of the mode of seasonal change in the percent body fat in the three different populations, where that percent body fat was relatively lower in the hot season than in the colder seasons. (In Chiang Mai, the hotter season is Apr to May while the colder season is Jan to Feb, see Table 1.)

Regarding body weight, there was a significant seasonal change in the Polish participants’ body weight, which was in the same order as that in the percent body fat. There were, in contrast, no significant seasonal changes in those of Japanese and Thai participants, although there were significant seasonal changes in the percent body fat. This could be explained by the fact that we measured the participants’ body weight including their clothes (without their outer wear). The body weight without clothes of Japanese and Thai participants were lower than those of Polish participants, so that the even a small seasonal change in the weight of the clothes worn would affect body weight. We are now re-investigating the seasonal change in body composition, including the percent body fat, body weight and muscular mass, of Japanese and Thai participants wearing the same clothes for every examination throughout a year.

### Seasonal variation in macronutrient intake

As summarized in Table 3, the results of macronutrient intakes of the two participant groups reflected characteristics of Japanese and Polish dietary habits. Polish participants take in more energy, protein and fat than Japanese participants do, while Japanese participants take in more carbohydrates than Polish participants do. Detailed discussion about the present food intake survey in Japanese and Polish participants will be published elsewhere, using food intake records of 100 Japanese and 111 Polish participants. The points discussed here are the seasonal changes in fat and carbohydrate intake, in addition to those in the C/F ratio. As shown in Table 3, there was no significant seasonal variation in the C/F ratio in Japanese participants. Owaki *et al*. [27] reported similar results, with no significant seasonal variation in total energy intake or in the ratio of energy intake from protein, fats and carbohydrates (similar to C/F ratio). In Japan, the disappearance of a seasonal variation in food intake has accelerated in recent years due to rapid development of the food transportation system and food production control including intensive culture by house and frozen storage.

In Polish participants, the C/F ratio in Apr to May was significantly lower than that in Jul to Aug, which reflected a significant decrease in the percentage of total energy from carbohydrate intake, and a non-significant increase in energy from fat intake Apr to May. This increase in fat intake in spring (and in winter) was also reported by Przysiężna and Banachowicz [28] with female students of the Wroclaw University of Economics. Their percentage of fat intake in the total energy intake was 32 % in winter, 32 % and 30 % in autumn. This increase in fat intake may be related to the effect of high-fat diet on thermal acclimation proposed by Yoshimura *et al*. [29] as a metabolic adaptation to cold temperatures in winter. Therefore, we should take into account the effect of ingestion of food rich in fat on the increase in the percent body fat in the colder seasons in Polish participants. These facts may be reflected in the relatively higher correlation coefficient within participants (although not significant) between the fat intake (as independent variable) and the percent body fat (dependent variable) in Table 5.

## Conclusions

We found that there were different seasonal variations in the fasting RQ values in the three different populations. There was a significant seasonal variation in the fasting RQ values in Japanese participants, while those in Polish and Thai participants were non-significant. In addition, we found that there were significant seasonal changes in the percent body fat in the three populations but we could not find any significant correlation between the fasting RQ values and the percent body fat.

## Abbreviations

AUDC: Amount of unabsorbed dietary carbohydrates; FFQ: Food frequency questionnaire; RQ: Respiratory quotient; VE: expiratory minute volume.

## Competing interests

The authors have no conflict of interest to declare.

## Authors’ contributions

YS was a general coordinator and did the study design. MW, JJ, PL, LM and NH designed the study and involved in data collection, data interpretation and result analysis. TM, JB and SK were involved in data collection, data interpretation, and result analysis and literature search. All authors contributed in preparing and are responsible for final editing and approval of the manuscript.
